# Editorial: Innovative strategies for the discovery of new therapeutic targets in cancer treatment

**DOI:** 10.3389/fonc.2026.1907345

**Published:** 2026-06-29

**Authors:** Fabrizio Carta, Concettina La Motta, Raivis Zalubovskis, Ilaria D’Agostino

**Affiliations:** 1 Neurofarba Department, University of Florence, Florence, Italy; 2 Department of Pharmacy, University of Pisa, Pisa, Italy; 3 National Institute of Research and Innovation, Riga, Latvia; 4 Institute of Chemistry and Chemical Technology, Faculty of Natural Sciences and Technology, Riga Technical University, Riga, Latvia

**Keywords:** anticancer drug discovery, biomarker discovery, cancer stem cells, cancer therapy, drug resistance, precision oncology, therapeutic targets, translational research

Cancer remains one of the most complex medical challenges, not only because of its high incidence and mortality but also because of its remarkable biological heterogeneity and adaptability. Accordingly, its management can no longer rely solely on traditional treatments based on the indiscriminate suppression of rapidly proliferating cells. Despite major advances in targeted therapy, immunotherapy, and precision oncology, long-lasting clinical benefit remains limited by disease recurrence, metastasis, and drug resistance. These processes are sustained by tumor heterogeneity, the dynamic interplay with tumor microenvironment (TME), and the persistence of cell subpopulations endowed with stem-like properties, plasticity, and adaptive survival mechanisms, commonly referred to as cancer stem cells (CSCs). Anticancer drug discovery is further complicated by interconnected tumor determinants such as hypoxia, metabolic reprogramming, pH regulation, aldehyde detoxification, epigenetic remodeling, DNA damage responses, and immune escape. Hypoxic tumors, for example, exploit carbonic anhydrases (CAs) IX and XII to regulate extracellular acidification, invasion, metastasis, and CSC fitness, making these tumor-associated isoforms highly attractive therapeutic targets (1, 2). Likewise, aldehyde dehydrogenases (ALDHs), particularly ALDH1A3, have recently emerged as functional markers and drivers of stemness, invasiveness, and resistance, encouraging the development of isoform-selective inhibitors to impair aggressive tumor phenotypes (3, 4).

Against this background, the Topic Editors recognize that an effective cancer therapy requires an integrated advancement of target discovery, biological validation, and rational drug design.

The Research Topic was therefore conceived to provide a broad platform for studies identifying novel cancer-related molecular determinants and translating these findings into biomarkers, experimental models, and innovative therapeutic interventions. It brings together 23 contributions, predominantly original research articles complemented by reviews, systematic analyses, case reports, and a commentary. Most contributions originate from Chinese institutions, with additional participation from research groups based in Europe and India. Collectively, they reflect the multidisciplinary nature of anti-cancer drug discovery, encompassing molecular-target and biomarker discovery, drug repurposing, combination therapy, advanced preclinical models, and translational clinical research. Despite the diversity of cancer types and experimental approaches employed, these contributions converge on a shared translational pathway transforming the identification of cancer-specific molecular vulnerabilities into validated and therapeutically exploitable applications.

Several studies identify molecular determinants of tumor progression. In diffuse large B-cell lymphoma (DLBCL), Qian and Yang showed that Transmembrane Emp24 Domain Containing 2 (TMED2) silencing suppressed tumor-cell growth through G0/G1 arrest, supporting TMED2 as a potential therapeutic target. In the same disease settings, Zhao et al. associated high protein kinase C delta (PKCδ) expression with poorer five-year progression-free and overall survival. In triple-negative breast cancer (TNBC), Wang et al. identified the H3K9 methyltransferase SUV39H2 as an upregulated promoter of malignant phenotypes, whereas Li et al. explored the role of palmitoylation of CD82, a metastasis suppressor, showing that CD82 palmitoylation is a mechanism of enhancing sensitivity to gefitinib and doxorubicin through apoptosis regulation. Chloride Intracellular Channel 6 (CLIC6) was also proposed as a pan-cancer biomarker and potential therapeutic target with experimental validation in breast cancer by a research group from Urumqi, China. Collectively, these studies demonstrate how alterations in protein expression, epigenetic regulation, and post-translational modification can reveal previously underexplored molecular dependencies associated with cancer progression and therapeutic response.

Thus, the search for useful molecular determinants was further extended through integrative bioinformatics tools and broader analyses of signaling pathways across cancer types. An integrated multi-omics bioinformatics analysis was reported by Shang et al. to underscore the pivotal role of the procollagen-lysine 2-oxoglutarate 5-dioxygenase (PLOD) family in the pathogenesis of clear cell renal cell carcinoma (ccRCC), whereas a perspective article by authors from the University of Queensland, Australia, examined the role of oncostatin M and its receptor (OSMR) across hematological and non-hematological malignancies.

Beyond identifying potential therapeutic targets, several contributions examined whether molecular alterations could also support prognostic assessment and patient stratification. Researchers from the Affiliated Hospital of Guizhou Medical University, China, identified and proposed three prognostic DNA methylation-related genes (MDM-RGs) for colorectal cancer (CRC). This article was followed by a commentary by Jiang et al. expanding their interpretation and biological relevance. These contributions highlight how molecular signatures may acquire clinical relevance when integrated into prognostic signatures and are well interpreted in the broader biological context of the disease.

DNA damage response pathways emerged as central molecular interfaces between genomic instability, immunity, and therapeutic vulnerability. Zhang et al. presented a DNA Damage Repair (DDR)-Immune Fitness score for small cell lung cancer (SCLC) to support patient stratification and guide rational combinations of poly-ADP-ribose polymerase (PARP) inhibition and immunotherapy. Su et al. reviewed the function of Ataxia telangiectasia and Rad3-related kinase (ATR) in solid tumors, highlighting its relevance as a therapeutically actionable target through pharmacological inhibition. Together, the studies by Zhang and Su position DNA damage response pathways at the interface of target discovery, biomarker development, and rational combination therapy.

Consistent with this translational perspective, another main theme of this Research Topic is the development of therapeutic strategies aimed at overcoming drug resistance. Xu et al. reviewed pharmacological strategies for reversing immune-checkpoint-inhibitor resistance in non-small-cell lung cancer (NSCLC), providing a framework for restoring immune responsiveness, whereas Liang et al. discussed phase 3 randomized controlled trials (RCTs) to compare KRASG12C inhibitors (KGIs) with respect to conventional chemotherapy in KRASG12C-mutated NSCLC. Two case reports addressed complex treatment decisions in lung adenocarcinoma. Researchers from the Weifang People’s Hospital, China, reported the coexistence of an NTRK2 fusion and EGFR mutations in advanced lung adenocarcinoma, whereas Zhang et al. reported sequential almonertinib treatment following osimertinib-induced grade 2 interstitial lung disease in early-stage Epidermal Growth Factor Receptor (EGFR) L858R-mutant lung adenocarcinoma.

Similar challenges related to resistance and prognostic stratification also emerged in hematological malignancies, particularly myeloid leukemia. Peng et al. reviewed the molecular basis of sorafenib resistance in FMS-like tyrosine kinase 3 (FLT3)-mutated acute myeloid leukemia (AML), whereas in the same disease, Yan et al. developed the machine-learning-based prognostic model named “malignant leukemia marker gene prognostic signature” (MLAPS), integrating transcriptomic, prognostic, and TME data from multicenter cohorts.

Taken together, these contributions highlight that the clinical exploitation of molecular targets requires not only the selection of effective agents, but also an understanding of cancer biology and patient-specific molecular contexts.

Moving from molecular characterization to therapeutic intervention, the translational potential of combination strategies was exemplified by a research group from the Zhongshan Hospital of Traditional Chinese Medicine, who investigated microwave ablation followed by fruquintinib, a selective Vascular endothelial growth factor receptor (VEGFR) 1–3 inhibitor, and tislelizumab, an anti-PD-1 monoclonal antibody, in patients with metastatic colorectal cancer (mCRC) and liver metastases (China Clinical Trial Center, ChiCTR2200058323). The triple-combination strategy improved overall survival, supporting the potential synergy among local tumor ablation, antiangiogenic therapy, and immune-checkpoint blockade.

The collection also renews interest in metal-based compounds, small-molecule inhibitors, and repurposing strategies. Researchers from Czechia developed ruthenium-enhanced curcumin complexes that displayed anticancer and anti-inflammatory activity in head and neck cancer (HNC) models. While this study explored the chemical modification of a natural-product-derived scaffold, curcumin, Zhou et al. pursued a protein-based strategy by developing TPCH, a cell-penetrating fusion protein obtained by combining the transactivator of transcription (TAT) peptide with exon 7 of the liver-related putative tumor suppressor (LPTS)/PIN2 interacting telomerase inhibitor 1 (PinX1). The researchers showed that TPCH is able to inhibit telomerase-positive cancer models *in vitro* and showed antitumor activity *in vitro* and in liver cancer patient-derived xenograft models *in vivo*. Complementing these novel therapeutic approaches, drug repurposing stands as a valuable opportunity to accelerate the identification of clinically applicable anticancer agents. Specifically, researchers from the Netherlands investigated the potential antitumor activity of the antipsychotic penfluridol in preclinical and “near-patient” advanced prostate cancer (PCa) models with the aim of drug repositioning.

Furthermore, the translational scope of the Research Topic was broadened by two reviews addressing advanced patient-oriented experimental models and the biological mechanisms underlying metastatic persistence. In particular, Wang et al. authored a review on Organoid-on-a-Chip technology in anticancer application, emphasizing the potential of patient-oriented platforms to model tumor microenvironment complexity and advance precision cancer therapy, whereas researchers from the Vellore Institute of Technology, India, reviewed the metastatic organotropism and tumor dormancy, addressing the persistence and reawakening of disseminated tumor cells.

Despite these advances, translating emerging therapeutic targets and anticancer agents into clinically effective interventions remains highly challenging. Indeed, candidate biomarkers, molecular determinants, and therapeutic strategies still require robust validation in clinically representative cohorts, while bioinformatics-derived signatures must demonstrate reproducibility and prognostic value beyond established markers. At the same time, advanced experimental models will be essential for assessing tumor heterogeneity, TME interactions, and treatment responses under conditions that more faithfully represent human physiology and disease.

Overall, the contributions collected in this Research Topic show that the discovery of effective anticancer therapies requires an iterative and interdisciplinary process. It requires the integration of molecular vulnerability identification, biomarker development, and patient stratification along with rational therapeutic designs, computational prioritization, mechanistic validation, and advanced experimental modeling. The convergence of these complementary approaches will be essential to translate biological insights into more precise, effective, and patient-centered anticancer strategies.
